# Tanshinone I, tanshinone IIA, and cryptotanshinone: key bioactive components modulating vascular smooth muscle cell function

**DOI:** 10.3389/fphar.2025.1688338

**Published:** 2025-10-09

**Authors:** Jixin Li, Wenru Wang, Hongbo Huang, Ruiling Zhou, Zhenyu Yang, Zhijie Cui, Zelong Niu, Shengnan Shi, Peili Wang

**Affiliations:** Chinese Academy of Traditional Chinese Medicine Xiyuan Hospital, Beijing, China

**Keywords:** cardiovascular diseases, vascular smooth muscle cells, tanshinone I, tanshinoneIIA, Cryptotanshinone, sodium tanshinone IIA sulfonate

## Abstract

Cardiovascular diseases (CVDs) remain the leading cause of mortality among non-communicable diseases worldwide. Vascular smooth muscle cells (VSMCs), as the predominant cellular component of the tunica media, are essential for maintaining vascular homeostasis through phenotype-dependent regulation of vascular tone, blood pressure, and hemodynamics. Under pathological conditions such as hypoxia or inflammation, VSMCs undergo phenotypic switching from a contractile to a synthetic state. This transition is characterized by excessive proliferation, migration, and pro-inflammatory secretion, all of which contribute to the progression of atherosclerosis and restenosis. Tanshinones, bioactive diterpenoid compounds isolated from *Salvia miltiorrhiza*, exert cardioprotective effects through their anti-inflammatory, antioxidant, and VSMC-modulating activities. Increasing evidence suggests that tanshinones attenuate maladaptive VSMC behaviors by regulating calcium signaling, modulating programmed cell death pathways, and suppressing pro-inflammatory signaling cascades. These actions collectively inhibit phenotypic switching and mitigate vascular remodeling and plaque formation. Despite these advances, a comprehensive understanding of the precise molecular targets and signaling networks of tanshinones in VSMCs is still lacking. This review aims to integrate current evidence to delineate tanshinone-mediated VSMC regulatory mechanisms, provide mechanistic insights, and identify potential therapeutic targets for phenotype-directed interventions in CVDs.

## 1 Indroduction

Cardiovascular diseases (CVDs) represent a primary contributor to early deaths and a major driver of the escalating global healthcare burden (GBD 2019 Diseases and Injuries Collaborators, 2020). The latest Global Burden of Disease Study reveals a substantial increase in CVD prevalence, from 271 million cases in 1990 to 523 million cases in 2019, underscoring the pressing need for targeted interventions to combat this expanding public health challenge (Roth et al., 2020). The pathophysiological mechanisms underlying CVDs are multifaceted, encompassing atherosclerosis, extracellular matrix remodeling, vascular calcification, and immune cell infiltration (Libby, 2024). Vascular smooth muscle cells (VSMCs), constituting the vascular tunica media, critically regulate vascular homeostasis (Majesky and Weiser-Evans, 2022). Intraluminal lipoproteins and cytokines trigger VSMC migration and proliferation toward the intima, where extracellular matrix (ECM) synthesis and fibrous cap formation drive plaque development (Yu et al., 2024). Physiologically, VSMCs preserve a contractile phenotype necessary for vascular tone modulation and vasomotor control. Pathologically, stimuli including vascular injury, oxidative stress, or chronic inflammation induce synthetic phenotypic switching, enhancing proliferative and migratory capacities. This phenotypic switching represents a fundamental pathogenic driver of CVD progression (Zhao et al., 2024; Wang S. et al., 2024).

VSMCs display remarkable plasticity. In response to pathological stimuli such as vascular injury, infiltration of inflammatory cytokines, lipid accumulation, and oxidative stress, contractile VSMCs can dedifferentiate into a synthetic phenotype (Jiang and Qian, 2023; Khachigian et al., 2022). Synthetic VSMCs exhibit a flattened morphology, enlarged cell size, reduced contractile filaments, impaired contractile function, and enhanced proliferative capacity. These proliferative synthetic VSMCs can secrete matrix metalloproteinases (MMPs) and pro-inflammatory cytokines, thereby accelerating extracellular matrix remodeling and recruiting monocytes (Wang et al., 2020). Moreover, in extracellular environments with elevated calcium and phosphate concentrations, synthetic VSMCs release calcium-phosphate-rich vesicles, which can induce osteogenic transdifferentiation of VSMCs, leading to upregulation of osteogenic transcription factors and promoting spontaneous vascular calcification (Reynolds et al., 2004). Single-cell sequencing studies have further revealed substantial heterogeneity among plaque-resident VSMCs, with certain subpopulations exhibiting stem cell–like properties and enhanced proliferative potential, potentially driving atherosclerotic plaque progression through clonal expansion (Gomez et al., 2013). Consequently, therapeutic strategies targeting VSMC plasticity offer significant clinical potential.

The regulation of VSMCs is complex and involves a network of molecular pathways. Natural medicines, particularly those with multi-target and multi-pathway regulatory effects, have demonstrated considerable potential in modulating VSMCs function. Tanshinones, a group of compounds derived from the Chinese herbal medicine *Salvia miltiorrhiza*, have been extensively studied for their cardiovascular benefits. These compounds include Tanshinone I (Tan I, C_18_H_12_O_3_), Tanshinone IIA (Tan IIA, C_19_H_18_O_3_), Dihydrotanshinone (C_18_H_14_O_3_), Cryptotanshinone (C_19_H_20_O_3_), Isotanshinone I (C_18_H_12_O_3_), Isotanshinone IIA (C_19_H_18_O_3_), and Isocryptotanshinone (C_19_H_20_O_3_) (Jiang et al., 2019). Among these, Tan I, Tan IIA, and Cryptotanshinone have garnered significant attention for their ability to improve cardiovascular function, as well as their anti-inflammatory, anti-tumor, and anti-oxidative properties (Shi et al., 2019; Wang J. et al., 2024; Wu et al., 2019). To overcome the limitation of poor water solubility of tanshinones, the sulfonated derivative of Tan IIA, Sodium tanshinone IIA sulfonate (STS), has been developed and shown to be effective in the treatment of CVD (Wei et al., 2024).

Research into the pharmacodynamics and molecular mechanisms of natural active compounds is currently a major focus in cardiovascular pharmacology. Recent studies suggest that tanshinones may regulate VSMCs function, positioning them as potential therapeutic agents for CVD. However, a systematic understanding of their precise molecular mechanisms and specific molecular targets remains lacking. This review explores the molecular mechanisms by which tanshinones modulate VSMC function, thereby elucidating the basis for their cardiovascular protective effects and establishing a scientific foundation for their therapeutic application in CVD.

## 2 Mechanisms of tanshinones in regulating VSMCs function

**FIGURE 1:**
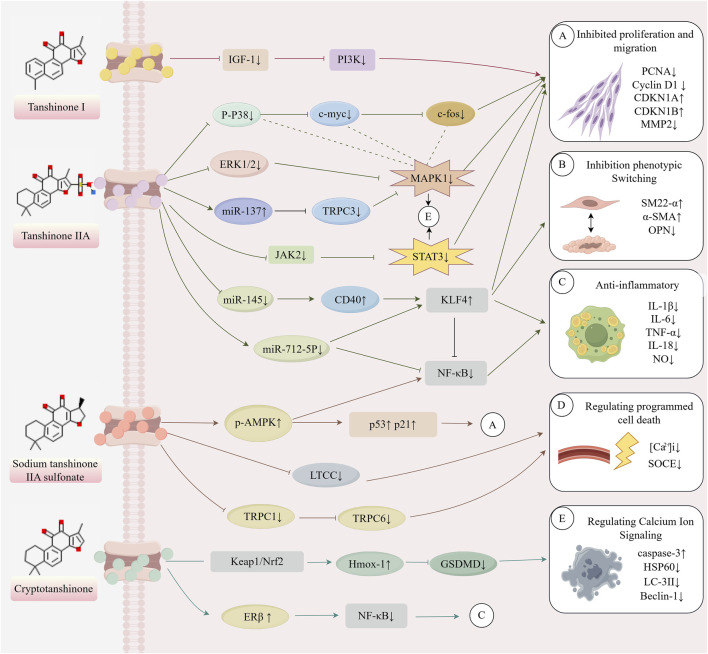
Mechanism of action of tanshinone in regulating VSMCs (This image was originally created by Figrdraw, ID: YPIWO04099).

The healthy arterial structure consists of three distinct layers (Majesky and Weiser-Evans, 2022): (1) the tunica intima, a single endothelial cell layer lining the bloodstream; (2) the tunica media, composed of VSMCs and layers of elastic fibers, including elastin and collagen, which provide elasticity and strength to the blood vessels; and (3) the tunica adventitia, made up of adipocytes, fibrous connective tissue, and extracellular matrix, which provide structural support, nutrition, and innervation. The elasticity of arteries primarily arises from the active contraction of VSMCs and the passive recoil of the elastic lamellae formed by collagen and elastin fibers. As a result, contractile VSMCs play a crucial role in regulating vascular diameter, blood pressure, and blood flow distribution (Yamin and Morgan, 2012). In large arteries, VSMCs maintain a contractile state to preserve the normal shape of arteries during ventricular systole and ejection, whereas in small resistance arteries, VSMCs are responsible for regulating blood flow distribution (Farina et al., 2020). The dysfunction or loss of normal VSMCs function represents a core pathological process in the onset and progression of various CVDs, including atherosclerosis, hypertension, and vascular restenosis. The mechanisms through which tanshinones regulate VSMCs function primarily include the inhibition of VSMCs proliferation and migration, suppression of VSMCs phenotypic switching, anti-inflammatory effects, regulation of programmed cell death, and modulation of calcium ion signaling. The synergistic interaction of these multiple targets and pathways forms an essential molecular foundation for the ability of tanshinones to alleviate VSMCs dysfunction and exert cardiovascular protective effects. Table 1 presents the references cited in this study, while Figure 1 illustrates the mechanism by which tanshinones regulate VSMC function.

**TABLE 1:** Experimental data on tanshinone-mediated VSMC regulation.

Reference	Compound	Experimental model	Concentration	Duration	Setting	Key effects	Molecular markers & pathways	Target disease	Proliferation/migration Cell seeding density
Lu et al. (2019)	Tan IIA	SD rat aortic VSMCs + Ang II	3.4, 17.0, 34.0 μmol/L	24 h, 48 h	*in vitro*	Inhibited proliferation; Promoted apoptosis; Increased autophagy	LC3-II↓, Beclin-1↓, p-p38↓, c-Myc↓, c-Fos↓, MAPK↓	–	5 × 10^3^ cells/well, -
Li et al. (2023)	Tan IIA	Human VSMCs + ox-LDL	8.5, 17.0, 34.0 μmol/L	24 h	*in vitro*	Inhibited proliferation and migration	miR-137↑, TRPC3↓, PCNA↓	Atherosclerosis	1 × 10^4^ cells/well, -
Lu et al. (2018)	Tan IIA	SD rat aortic VSMCs + AGEs	10 μmol/L	48 h	*in vivo*	Inhibited proliferation and migration	p-ERK1/2↓, MAPK↓	–	1 × 10^4^ cells/well, 1 × 10^4^ cells/well
Li X. et al. (2020)	Tan IIA	Human VSMCs + Hcy	0, 0.1, 1, 10 μmol/L	24 h	*in vitro*	Inhibited proliferation	miR-145↓, CD40↑, KLF4↑	–	-, 1 × 10^5^ cells/well
Lou et al. (2020)	Tan IIA	C57BL/6 mice + Carotid ligation	5 mg/kg	3 w	*in vivo*	Inhibited proliferation; Suppressed neointima; Attenuated remodeling	PCNA↓	Vascular damage	-, -
	Tan IIA	SD rat aortic VSMCs + PDGF-BB	1.0 μmol/L	12–30 h	*in vitro*	Inhibited proliferation, migration, phenotypic switching	MHC↑, Calponin↑, SM22α↑, Myocardin↑, SRF↑, Cyclin D1↓, CDKN1A↑, CDKN1B↑, KLF4↑	–	3 × 10^3^ cells/well, 1 × 10^6^ cells/well
Qin et al. (2020)	Tan IIA	C57BL/6 mice + Carotid ligation	10 mg/kg	21 days	*in vivo*	Suppressed neointimal hyperplasia; Anti-inflammatory	TNF-α↓, KLF4↑, IL-1β↓, IL-6↓	Vascular damage	-, -
	Tan IIA	Mouse aortic VSMCs + TNF-α	1.7, 3.4, 6.8 μmol/L	48 h	*in vitro*	Anti-inflammatory; Inhibited proliferation	TNF-α↓, KLF4↑, IL-1β↓, IL-6↓, miR-712-5p↓,NF-κB↓	–	1 × 10^4^ cells/well, -
Meng et al. (2019)	Tan IIA	SD rat aortic VSMCs + LPS	6.25–200 μmol/L	24 h	*in vitro*	Anti-inflammatory; Inhibited phenotypic switching	α-SMA↑, OPN↓, MCP-1↓, IL-6↓, TNF-α↓, iNOS↓, NO↓, ROS↓, TLR4↓, p-TAK1↓, NF-κB↓	–	5 × 10^3^ cells/well, 5 × 10^3^ cells/well
Chen et al. (2014)	Tan IIA	Wistar rat PASMCs + Hypoxia	1.7, 3.4, 17.0 μmol/L	24 h	*in vitro*	Reduced viability; Promoted apoptosis; Inhibited proliferation	Hsp60↓, Cleaved Caspase-3↑, JAK2↓, STAT3↓, Cx43↑	Hypoxia	-, -
Wu et al. (2014)	STS	SD rat aortic VSMCs + High Glucose	10 μmol/L	24 h	*in vitro*	Inhibited proliferation and migration	p-AMPK↑, Cyclin D1↓, p53↑, p21↑, NF-κB↓, MMP-2↓	Diabetic Vascular Disease	-, 4 × 10^4^ cells/well
Wei et al. (2024)	STS	db/db mouse aortic VSMCs + High Glucose	100 μmol/L	72 h	*in vitro*	Attenuated senescence; Anti-inflammatory	IL-1β↓, IL-18↓, A20↑, CAT↑, H_2_O_2_↓, NLRP3↓, Caspase-1↓, NF-κB↓	Diabetic Vascular Disease	-, -
(Wang et al., 2013)	STS	SD rats + Hypoxia + MCT	10 mg/kg	21 days	*in vivo*	Reduced Ca^2+^ influx; Improved hemodynamics	[Ca^2+^]i↓, SOCE↓, TRPC1↓, TRPC6↓	Pulmonary Hypertension	-, -
	STS	SD rat PASMCs + Hypoxia	0.1–25 μmol/L	24 h	*in vitro*	Inhibited proliferation, migration; Reduced Ca^2+^ influx	[Ca^2+^]i↓, SOCE↓, TRPC1↓, TRPC6↓	Hypoxia	-, 1 × 10^5^ cells/well
Zhang et al. (2018)	STS	SD rats + Hypoxia + MCT	30 mg/kg	21 days	*in vivo*	Reduced Ca^2+^ influx; Improved hemodynamics	[Ca^2+^]i↓, LTCC↓	–	-, -
	STS	SD rat PASMCs + Hypoxia	12.5 μmol/L	60 h	*in vitro*	Inhibited proliferation; Reduced Ca^2+^ influx	[Ca^2+^]i↓, LTCC↓	–	-, -
Wu et al. (2019)	Tan I	Human VSMCs + Ang II	0.625, 1.25, 2.5 μmol/L	24 h	*in vitro*	Inhibited proliferation	IGF-1↓, PI3K↓	–	5 × 10^3^ cells/well, -
Wang J. et al. (2024)	Cryptotanshinone	ApoE^−/−^ mice + Ang II	15, 50 mg/kg	28 days	*in vivo*	Inhibited phenotypic switching	α-SMA↑, SM22α↑	Abdominal aortic aneurysm	-, -
	Cryptotanshinone	SD rat aortic VSMCs + TNF-α	2.5–10 μmol/L	24 h	*in vitro*	Inhibited phenotypic switching; Anti-inflammatory; Attenuated pyroptosis	VCAM-1↓, MMP-2↓, MMP-3↓, MMP-9↓, α-SMA↑, SM22α↑, IL-1β↓, IL-6↓, CCL2↓, NLRP3↓, Nrf2/HO-1↑	–	1 × 10^4^ cells/well, -
Oche et al. (2016)	Cryptotanshinone	LPS + VSMCs A7r5	0.1, 1, 10 μmol/L	24 h	*in vitro*	Anti-inflammatory	ERβ ↑, iNOS ↓, NO ↓	–	5 × 103 cells/well, -

Drug concentrations are standardized to molarity (μmol/L) to enable direct cross-study comparison of pharmacological data. Conversions were performed using the formula: μmol/L = [μg/mL × 1,000]/Molecular Weight (g/mol), with compound-specific molecular weights applied to original values from each source study.

Tanshinones, the principal bioactive constituents of *S. miltiorrhiza*, have recently been shown to exert multifaceted cardiovascular protective effects. Accumulating evidence indicates that tanshinones effectively inhibit the proliferation and migration of VSMCs induced by ox-LDL and other stimuli, while preserving their contractile phenotype through the regulation of transcription factors such as KLF4. In addition, tanshinones modulate programmed cell death processes—including autophagy, apoptosis, and pyroptosis—thereby contributing to cellular homeostasis. Beyond these effects, their pronounced anti-inflammatory and antioxidant activities improve the vascular microenvironment. Tanshinones also influence VSMC contractile function and intracellular stability by regulating TRPC channels and calcium signaling pathways. This review summarizes recent advances in understanding how tanshinones regulate VSMC function. It highlights their molecular mechanisms across multiple pathways, including proliferation and migration, phenotypic switching, programmed cell death, inflammatory responses, and calcium signaling. These findings provide a theoretical foundation for advancing our knowledge of tanshinone pharmacology and for developing novel VSMC-targeted therapeutic strategies in cardiovascular disease. Table 1 presents the references cited in this study, while Figure 1 illustrates the mechanism by which tanshinones regulate VSMC function.

### 2.1 Inhibiting VSMCs proliferation and migration

Under physiological conditions, quiescent contractile VSMCs exhibit minimal proliferative activity and primarily contribute to regulating blood circulation by maintaining vascular tone and homeostasis (Lutgens et al., 1999). However, in response to vascular injury, their proliferative capacity increases abnormally. VSMCs migration is frequently coupled with proliferation, involving key biological processes such as the establishment of cell polarity, cytoskeletal remodeling, and the dynamic integration of microenvironmental signals (Afewerki et al., 2019). During initial vascular remodeling, injury, or CVD pathogenesis, local inflammatory mediators (e.g., TNF-α, IL-18, ox-LDL) induce VSMC migration toward the intima (Liu et al., 2016). These aberrantly proliferating/migrating VSMCs secrete excessive collagen, elastin, and MMPs, driving endothelial dysfunction and inflammatory microenvironment accumulation. Consequently, vessel wall thickening, luminal narrowing, and pathological remodeling occur, culminating in tissue hypoperfusion and functional impairment (Xin et al., 2024). Tan IIA exhibits both anti-inflammatory and antioxidant properties and plays a critical role in cardiovascular and inflammatory diseases (Fang et al., 2018; Chen and Xu, 2014). Moreover, accumulating evidence suggests that Tan IIA attenuates the progression of atherosclerosis by suppressing inflammation and oxidative stress in animal models (Wen et al., 2020; Chen et al., 2012). However, the precise mechanisms by which Tan IIA regulates ox-LDL–induced proliferation and migration of VSMCs remain largely unclear. Li (Li et al., 2023) treated ox-LDL-stimulated human VSMCs with Tan IIA at 10 μg/mL, demonstrating a dose-dependent inhibition of VSMCs proliferation and migration through regulation of the miR-137/TRPC3 axis. Similarly, Yao Li (Li Y. et al., 2020) confirmed that Tan IIA can inhibit homocysteine (Hcy)-induced VSMCs proliferation by upregulating miR-145 and downregulating CD40. Moreover, signaling pathways such as the ERK1/2 MAPK pathway (Lu et al., 2018) and the AMPK/p53/p21 pathway (Wu et al., 2014) may also represent potential targets for Tan IIA action. Additionally, Tan I has shown potential in regulating VSMCs function. Wu (Wu et al., 2019) found that Tan I dose-dependently inhibits the IGF-1R/PI3K signaling pathway, suppressing the expression of VSMCs proliferation-related proteins, including CDK4, cyclin D3, and cyclin D1, thereby reducing VSMCs proliferative capacity. These studies collectively provide compelling evidence supporting the role of tanshinones in antagonizing VSMCs proliferation and migration.

### 2.2 Inhibiting VSMCs phenotypic switching

VSMCs exhibit remarkable plasticity. In response to pathological stimuli such as vascular wall injury, growth factor stimulation, inflammatory cytokine infiltration (e.g., TNF-α, IL-6, IL-18), lipid accumulation, and oxidative stress, contractile VSMCs undergo dedifferentiation, transitioning into synthetic VSMCs (Jiang and Qian, 2023; Khachigian et al., 2022). Synthetic VSMCs exhibit a flattened morphology, with increased cell size, reduced myofilaments, and diminished contractile function. Notably, following injury repair, synthetic VSMCs can revert to the contractile phenotype (Yu et al., 2024; Wang et al., 2023; Zhang et al., 2025). In the treatment of vascular diseases, tanshinone IIA has been shown to attenuate atherosclerotic calcification primarily through the inhibition of oxidative stress (Tang et al., 2007). However, whether tanshinone IIA plays a pivotal role in regulating VSMC phenotypic switching and pathological vascular remodeling remains largely unclear. Through a combination of *in vivo* and *in vitro* experiments, Lou (Lou et al., 2020) demonstrated that Tan IIA regulates VSMCs phenotypic switching by enhancing the expression of KLF4. This results in the inhibition of VSMCs proliferation, the induction of differentiation, and the modulation of pathological vascular remodeling. Wang J. et al. (2024) demonstrated that 28-day cryptotanshinone infusion in Ang II-induced ApoE^−/−^ mouse aortic aneurysm models attenuated reductions in VSMC contractile markers (α-SMA, SM22α). Synthetic VSMCs may transdifferentiate into diverse pathological phenotypes under specific stimuli, notably osteoblast-like VSMCs–key drivers of vascular calcification that increase arterial stiffness and reduce compliance (Hortells et al., 2018). These findings collectively demonstrate tanshinones’ capacity to modulate pathological VSMC plasticity.

### 2.3 Regulating programmed cell death

Programmed cell death plays a crucial role in cellular pathophysiology, as it helps maintain cellular homeostasis by regulating cell survival and death. The common forms of programmed cell death include autophagy, apoptosis, and pyroptosis (Kari et al., 2022). While moderate autophagy is essential for maintaining VSMCs phenotype and contractile function, excessive autophagy can adversely affect cell survival and promote the transition of VSMCs from a contractile to a synthetic phenotype, thereby diminishing VSMCs contractile function (Li et al., 2019; He et al., 2022). Previous studies have demonstrated that Tan IIA can inhibit cardiomyocyte apoptosis via suppression of the ERK1/2 signaling pathway (Zhang et al., 2012). In addition, Tan IIA has been reported to regulate programmed cell death in cancer cells through modulation of multiple signaling pathways (Won et al., 2010; Yun et al., 2013). Lu (Lu et al., 2019) demonstrated that Tan IIA (5–10 μg/mL) suppresses Ang II-induced VSMC proliferation and dysregulated autophagy via MAPK pathway downregulation, concurrently promoting apoptosis *in vitro*. Complementarily, Chen (Chen et al., 2014) reported Tan IIA modulation of Hsp60, caspase-3, and connexin 43 (Cx43) expression through JAK2/STAT3 signaling, thereby inducing apoptosis in hypoxic PASMCs. Notably, pathological pyroptosis in VSMCs critically drives abdominal aortic aneurysm pathogenesis. Wang J. et al. (2024) observed that cryptotanshinone can activate the transcription of Nrf2 target genes in TNF-α-induced thoracic aortic VSMCs from Sprague-Dawley rats. This activation prevents NLRP3 inflammasome activation and GSDMD-mediated pyroptosis, thereby alleviating VSMCs inflammation and preserving the contractile phenotype of VSMCs.

### 2.4 Anti-inflammatory effects

The arterial wall is composed of multiple layers that contain a significant number of immune cells. Upon lipid accumulation and vascular injury, the number of immune cells increases, leading to the secretion of large amounts of pro-inflammatory cytokines. This secretion promotes the formation of an inflammatory microenvironment (Blagov et al., 2023). Inflammatory stimuli trigger VSMC transition from quiescent contractile to synthetic phenotypes. These activated synthetic VSMCs amplify inflammatory infiltration, accelerating vascular pathogenesis (Wu et al., 2019). Consistent with previous reports, Tan II exhibits potent anti-inflammatory properties in a variety of pathological conditions, including inflammatory bowel disease (Li X. et al., 2020), cancer (Xie et al., 2025), myocardial infarction (Ren et al., 2010), and diabetic (Liu et al., 2024). Therefore, it may also possess the potential to modulate the inflammatory milieu of VSMCs. Meng (Meng et al., 2019) demonstrated in LPS-stimulated rat aortic VSMCs that Tan IIA attenuates MCP-1, IL-6, TNF-α, and NO production via TLR4/TAK1/NF-κB axis suppression. Concurrently, Tan IIA potentiates α-SMA expression while inhibiting pathological phenotypic switching. Yan (Qin et al., 2020) further established that Tan IIA downregulates miR-712-5p, upregulating (KLF4) to suppress NF-κB activation, thereby reducing neointimal hyperplasia and VSMC inflammation. Another study (Wei et al., 2024) demonstrated that sodium STS suppresses NLRP3 inflammasome activation in high glucose-exposed VSMCs through NF-κB signaling inhibition, thus ameliorating inflammatory microenvironments and attenuating vascular aging. Interestingly, the study by Oche (Oche et al., 2016) revealed that cryptotanshinone possesses phytoestrogen-like properties. Specifically, cryptotanshinone binds to estrogen receptor beta (ERβ), which in turn suppresses LPS-induced expression of inducible nitric oxide synthase (iNOS) and reduces excessive nitric oxide (NO) production. Through this mechanism, cryptotanshinone exerts notable anti-inflammatory effects in vascular smooth muscle cells.

### 2.5 Regulating calcium ion signaling

In pulmonary artery smooth muscle cells (PASMCs), elevated intracellular Ca^2+^ ([Ca^2+^]i) – predominantly mediated by store-operated calcium entry (SOCE) through TRPC channels–critically regulates cellular contraction and growth (Liu et al., 2003; Wang et al., 2004; Wang et al., 2006). Consequently, modulating Ca^2+^-dependent contractility represents a pharmacological target for vasodilation. Recent studies have demonstrated that STS exerts protective effects against hypoxic pulmonary hypertension, including reductions in pulmonary arterial pressure, pulmonary arterial wall thickness, and right ventricular hypertrophy. These beneficial effects are thought to be partially mediated through the regulation of intracellular Ca^2+^ homeostasis in PASMCs (Huang et al., 2009). Wang (Wang et al., 2013) emonstrated that STS suppresses hypoxia-induced SOCE and basal [Ca^2+^]i elevation in chronic hypoxic pulmonary hypertension models and hypoxic PASMCs by downregulating TRPC1/TRPC6. This inhibition consequently attenuated right ventricular hypertrophy and pathological pulmonary vascular remodeling. Furthermore, a 2018 study (Zhang et al., 2018) found that STS can directly block voltage-gated L-type calcium channels (LTCCs), thereby regulating [Ca^2+^]i and alleviating vascular tone. Both studies noted a concentration-dependent effect of STS and a non-potassium channel-dependent pathway. These findings provide a novel paradigm for the development of vasodilatory therapies targeting the calcium signaling network in VSMCs.

## 3 Conclusion and perspective

In summary, tanshinones exhibit comprehensive multi-target and multi-pathway effects in regulating VSMCs function, offering new insights into drug development for CVD treatment. Research has shown that tanshinones can effectively inhibit abnormal VSMCs proliferation and migration, block pathological phenotypic switching, and regulate calcium signaling, programmed cell death, and the inflammatory microenvironment. Collectively, these actions alleviate vascular wall remodeling, plaque formation, and luminal stenosis, highlighting their potential in combating CVDs such as atherosclerosis and myocardial infarction through the modulation of VSMCs homeostasis. Based on existing research, the application of tanshinones in VSMCs and their potential mechanisms for treating CVDs have primarily focused on tanshinone IIA and STS. Tanshinone II (Tan II) exhibits substantial potential in modulating a wide range of VSMC functions, positioning it as a promising candidate for broadly regulating VSMC activity to confer cardiovascular protection. In contrast, Sodium Tanshinone IIA Sulfonate (STS) demonstrates unique advantages in modulating calcium signaling, markedly attenuating right ventricular hypertrophy and pathological pulmonary vascular remodeling under hypoxic conditions, and improving pulmonary arterial wall thickness. In contrast, investigations into Tan I and cryptotanshinone remain relatively limited. Nevertheless, previous studies have demonstrated that cryptotanshinone exerts cardioprotective effects through the regulation of multiple cellular pathways, including oxidative stress, autophagy, mitochondrial function, and inflammatory responses (Zheng et al., 2024). For example, cryptotanshinone has been shown to enhance cell viability by downregulating the ERK and NF-κB pathways, upregulating the anti-apoptotic gene Bcl-2, inhibiting the production of reactive oxygen species (ROS) and malondialdehyde (MDA), and activating the MAPK3 pathway to suppress caspase-3 cleavage. Collectively, these actions mitigate myocardial oxidative stress, inhibit cardiomyocyte apoptosis, and provide protection against myocardial ischemia-reperfusion injury (Mao et al., 2021; Zhang et al., 2021). Similarly, studies have confirmed that Tan I significantly improves survival in cardiomyocyte models subjected to oxidative stress, alleviates intracellular oxidative damage, and suppresses ROS release (Ke et al., 2025). Furthermore, Tan I restores electrocardiographic abnormalities in murine models of myocardial infarction, reduces infarct size, and inhibits cardiac fibrosis. Although the specific roles of these compounds in VSMCs require further clarification, their demonstrated cardioprotective effects highlight considerable therapeutic potential and warrant future investigation within the field of vascular medicine.

The principal innovations of this study are as follows. First, we systematically integrated current evidence on the multi-faceted mechanisms by which tanshinones modulate VSMCs function, thereby constructing a comprehensive regulatory network that encompasses phenotypic switching, programmed cell death, calcium homeostasis, and inflammatory responses. This integration enhances the understanding of the multi-target effects of natural compounds on VSMCs. Second, by standardizing pharmacological data to molar concentrations and integrating cross-species experimental evidence, this study demonstrates that tanshinones attenuate pathological VSMC remodeling through key signaling axes—such as KLF4/miR-137–TRPC3, TLR4/NF-κB, and Nrf2/HO-1—and highlights their novel role in fine-tuning the balance between autophagy and pyroptosis. Furthermore, we summarize the emerging roles of understudied tanshinones, including Tan I and Cryptotanshinone, which exhibit protective effects in models of cardiovascular injury and show promise as potential therapeutic agents, warranting further investigation.

However, several limitations remain in current research. First, the regulatory weight of tanshinones on specific targets and the synergistic mechanisms of cross-pathways remain unclear. For instance, the hierarchical relationships between targets such as KLF4 and TRPC3 in phenotypic switching require further investigation. Second, while the issue of low bioavailability of natural tanshinones has been partially addressed by the development of STS, there is still considerable room for improvement in targeted delivery systems. Third, most conclusions are based on cellular and animal models, with a lack of large-scale clinical evidence to verify their long-term cardiovascular protective effects.

Future investigations should prioritize the following research avenues: (1) Integrating single-cell sequencing and proteomics technologies to systematically map the signaling network regulated by tanshinones in VSMCs, and to identify their core targets. (2) Utilizing synthetic biology techniques to modify tanshinone structures or develop nanocarriers to enhance tissue targeting and bioavailability. (3) Conducting multicenter clinical trials to evaluate the effects of tanshinone preparations on major adverse cardiovascular events in CVD patients, with particular emphasis on their long-term impact on vascular intimal hyperplasia and myocardial remodeling. By advancing the “mechanism-translation-clinical” chain through interdisciplinary collaboration, tanshinones hold promise as key candidate molecules for the next-generation of cardiovascular protective drugs.
